# Automatic 3D Tracking of Liver Metastases: Follow-Up Assessment of Cancer Patients in Contrast-Enhanced MRI

**DOI:** 10.3390/bioengineering12080874

**Published:** 2025-08-12

**Authors:** Sophia Schulze-Weddige, Uli Fehrenbach, Johannes Kolck, Richard Ruppel, Georg Lukas Baumgärtner, Maximilian Lindholz, Isabel Theresa Schobert, Anna-Maria Haack, Henning Jann, Martina Mogl, Dominik Geisel, Bertram Wiedenmann, Tobias Penzkofer

**Affiliations:** 1 Department of Radiology, Charité Universitätsmedizin Berlin, 13353 Berlin, Germany; uli.fehrenbach@charite.de (U.F.); johannes.kolck@charite.de (J.K.); richard.ruppel@charite.de (R.R.); georg.baumgaertner@charite.de (G.L.B.); maximilian.lindholz@charite.de (M.L.); isabel.schobert@charite.de (I.T.S.); anna-maria.haack@charite.de (A.-M.H.); dominik.geisel@charite.de (D.G.); tobias.penzkofer@charite.de (T.P.); 2 Division of Gastroenterology, Charité Universitätsmedizin Berlin, 10117 Berlin, Germany; henning.jann@charite.de (H.J.); bertram.wiedenmann@charite.de (B.W.); 3 Department of Surgery, Charité Universitätsmedizin Berlin, 10117 Berlin, Germany; martina.mogl@charite.de; 4 Berlin Institute of Health, 10178 Berlin, Germany

**Keywords:** liver, neoplasm metastasis, neuroendocrine tumors, magnetic resonance imaging, follow-up studies

## Abstract

Background: Tracking differential growth of secondary liver metastases is important for early detection of progression but remains challenging due to variable tumor growth rates. We aimed to automate accurate, consistent, and efficient longitudinal monitoring. Methods: We developed an automatic 3D segmentation and tracking algorithm to quantify differential growth, tested on contrast-enhanced MRI follow-ups of patients with neuroendocrine liver metastases (NELMs). The output was integrated into a decision support tool to distinguish between progressive disease, stable disease, and partial/complete response. A user study involving an expert group of seven expert radiologists evaluated its impact. Group comparisons used the Friedman test with post hoc analyses. Results: Our algorithm detected 991 metastases in 30 patients: 13% new, 30% progressive, 18% stable, and 18% regressive; the remainder were either too small to measure (15%) or merged with another metastasis in the follow-up assessment (6%). Diagnostic accuracy improved with additional information on hepatic tumor load and differential growth, albeit not significantly (*p* = 0.72). The diagnosis time increased (*p* < 0.001). All radiologists found the method useful and expressed a desire to integrate it in existing diagnostic tools. Conclusions: We automated segmentation and quantification of individual NELMs, enabling comprehensive longitudinal analysis of differential tumor growth with the potential to enhance clinical decision-making.

## 1. Introduction

Metastatic disease represents a significant challenge in the management of cancer, contributing to high mortality rates [1,2]. Due to the liver’s role in filtering blood from the gastrointestinal tract, it is a common site for secondary metastases [3,4]. The likelihood of developing liver metastases varies depending on the primary tumor location, with prevalences reaching up to 91% for some neuroendocrine tumors [5]. Efficient and accurate follow-up assessment of metastatic disease is a critical clinical factor for monitoring responses to therapy [6,7]. This includes tracking the differential growth of individual tumors for early detection of progression or recurrence, allowing timely adjustments to treatment plans.

The response evaluation criteria in solid tumors (RECIST1.1) guidelines assess tumors’ response to treatment by selecting up to two target lesions per organ. They are monitored in subsequent scans to classify disease status [8]. Non-target lesions are considered, although they are typically not measured. Hence, the selection of target lesions is highly important, especially for patients with mixed tumor responses [9]. Measuring each lesion individually would provide a more detailed and accurate assessment, but this is too time-consuming for routine clinical practice. Therefore, there is a clinical need for an accelerated evaluation of all individual metastases to enable a more accurate assessment.

The aim of this study is to develop a method to automatically track the differential neoplastic growth between baseline and follow-up imaging, using neuroendocrine liver metastases (NELMs) as an illustrative case. We used gadoxetic acid (Gd-EOB)-enhanced magnetic resonance imaging (MRI), as it exhibits the highest accuracy in detecting NELMs [10,11]. Our fully automated approach generates actionable information for radiologists. The usefulness of this information was assessed through a user study, testing its practical relevance and real-world applicability.

## 2. Materials and Methods

### 2.1. Patient Cohorts

For this study, two previously reported patient cohorts were included [12]. The first cohort was used for the training of the segmentation models and consisted of 220 Gd-EOB-enhanced (Primovist, Bayer, Berlin, Germany) MRI scans, which were retrospectively identified and manually segmented by radiologists with at least 5 years of experience in abdominal MRI. Scans were conducted between January 2015 and August 2018 at Charité University Hospital, Berlin, Germany, using a 1.5 T scanner (MAGNETOM Aera, Siemens Healthcare, Erlangen, Germany). The MRI examination protocols included a 3D T1-weighted gradient echo (GRE) sequence with fat saturation (FS) during the hepatobiliary contrast phase (HBP), using VIBE (volumetric interpolated breath-hold examination). The HBP sequence was acquired 20 min after the contrast administration.

The second cohort was used in the user study. It consisted of 30 patients who were discussed in our Multidisciplinary Tumor Board (MTB) between January 2019 and January 2020. The MTB decision of disease status served as the ground truth. The baseline and follow-up Gd-EOB MRI examinations were between 2 and 24 months apart (median: 6 months). Scans were conducted across five different institutional scanners, encompassing both 1.5 T and 3 T examinations. Each examination included a 3D T1-weighted GRE FS sequence during the hepatobiliary contrast phase. The HBP sequence was acquired between 10 and 20 min after contrast administration, depending on the allocated time of the examination and the liver function [13]. A detailed description of the cohorts can be found in a previous publication [12].

This study was conducted in accordance with the guidelines of the Declaration of Helsinki and was approved by the Institutional Review Board of Charité Berlin.

### 2.2. Automatic Lesion Detection and 3D Tracking

In a previous study, it was shown that nnU-Net, a self-configuring pipeline for advanced biomedical imaging segmentation [14], performs effectively in segmenting the liver and NELMs [12]. Therefore, we based our 3D tracking of individual metastases on the same approach by training two nnU-Net segmentation models. We employed the standard nnU-Net framework with its default self-configuring pipeline, without any modifications, utilizing the automatic hyperparameter optimization, training strategy, and post-processing as described in the original implementation [14].

The first model, which was trained to detect the liver, was used to register the baseline and follow-up images, and to exclude metastases beyond the liver’s boundaries. By aligning the segmented liver regions rather than focusing on the entire MRI images, more accurate and anatomically relevant registration results were achieved. The second segmentation model was trained to detect NELMs, distinguishing healthy tissue from metastatic areas.

In addition to the previous approach, where segmentation masks were only used to calculate the overall hepatic tumor load, we extended the method by separating individual metastases to analyze their specific growth dynamics. This was achieved by combining clusters of interconnected tumorous voxels to denote a single metastasis and assigning them a unified label. Then, metastases at baseline and follow-up were matched based on the shortest distance between their centers and the extent of their overlap, calculated with the Dice similarity coefficient [15]. For each lesion *i* at baseline and lesion *j* at follow-up, matching was performed using the following:

Euclidean distance: d(*i,j*) = $\surd {\left(ci\;-\;cj\right)}^{2}$, where *ci* and *cj* are the 3D centroids;

Dice overlap: Dice(*i,j*) = 2|Vi ∩ Vj|/(|Vi| + |Vj|), where *Vi* and *Vj* are the voxel sets.

**Figure 1:**
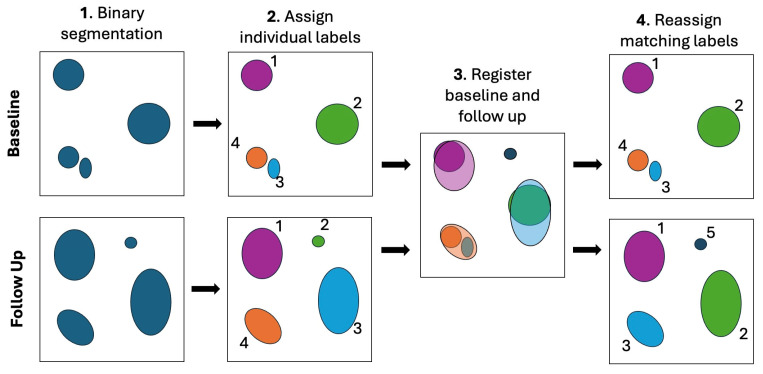
Graphical representation of the 3D tracking algorithm: For simplicity, the graphic is two-dimensional, but our method works with three-dimensional MRI volumes. In step 1, the binary segmentation masks are illustrated. They are automatically generated from the baseline and follow-up MRI with a deep learning-based segmentation model. In step 2, individual labels are assigned to all clusters of cancerous pixels for each MRI separately. The labels are indicated with the numbers 1–4 in this figure. In step 3, baseline and follow-up images are registered to align in the same space. Lastly, in step 4, the labels are reassigned according to the shortest distance and largest overlap. All lesions are now consistently labeled in baseline and follow-up. Lesion 5 is new and has no corresponding match in the baseline. Lesions 4 and 3 have merged and are now labeled as 3 in the follow-up. Lesion 4 will be marked as “merged” (refer to Figure 2 for a real example from the experiment).

**Figure 2:**
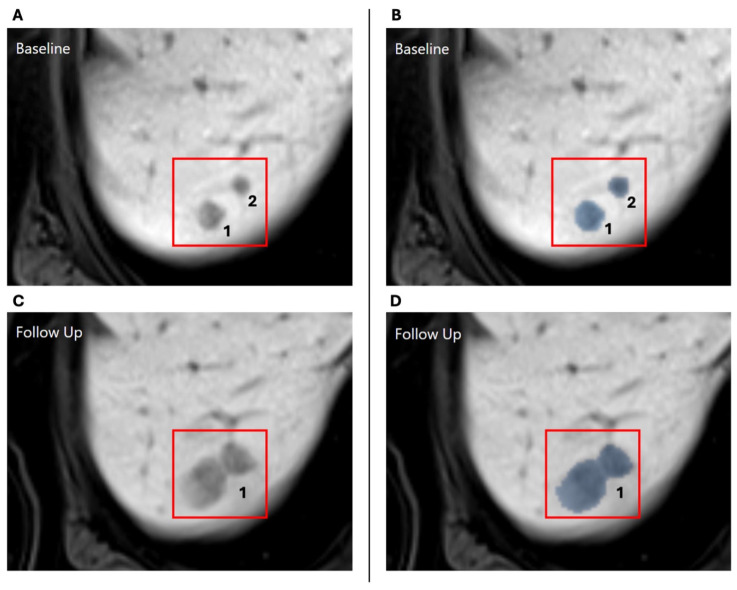
Example of a metastasis that is indicated as “merged”: (**A**) There are two separate metastases in the baseline MRI. (**B**) The baseline MRI is overlaid with the automatically generated segmentation mask highlighting the two metastases. (**C**) In the follow-up MRI, these metastases both grew such that they were very close together. (**D**) The follow-up MRI is overlaid with the automatically generated segmentation mask, which interprets the two metastases as one. The metastases will now be considered as “merged”.

See Figure 1 for a graphic representation of this process. Unmatched metastases can have three possible explanations: (1) a new metastasis appeared in the follow-up, (2) a metastasis from the baseline disappeared, or (3) a metastasis merged with another metastasis (see Figure 2 for an example).

From the new, consistently labeled segmentation masks, the differential growth of individual metastases was calculated. This included the volume, the largest axial diameter, and the differences between baseline and follow-up in absolute and relative numbers. With this information, each metastasis was categorized following the thresholds from the RECIST1.1 guidelines (see Table 1) [8].

### 2.3. Evaluation Study

An expert group of seven radiologists, with 1 to 12 years of experience in abdominal MRI (median 4 years), was assembled. They were tasked with the treatment response evaluation in 30 patients with NELMs based on baseline and follow-up MRI examinations. The possible answers were progressive disease, stable disease, and partial/complete response.

The study followed a within-subject repeated-measures design, consisting of three settings, each separated by at least a month. Both the sequence of the study settings and the order of the cases within each setting were randomized to avoid bias. For every case, the radiologists provided a treatment response evaluation, rated their certainty on a four-item Likert scale, and had the decision time recorded. After completing the study, they were asked to fill in a short survey regarding their opinion about the tool and to make comments. Details regarding the survey can be found in Appendix A.

**Figure 3:**
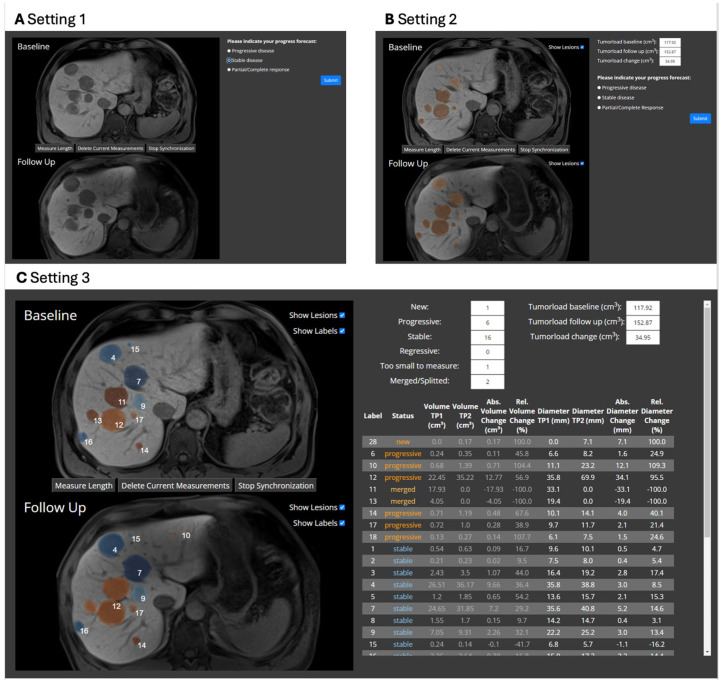
Different levels of information presented to the radiologists as part of the usability study: (**A**) Setting 1—Only baseline and follow-up MRI are shown. The MRI volumes are three-dimensional and can be scrolled through synchronized or unsynchronized, and length measurements can be made. Based on this, the radiologists make a treatment response evaluation, with the possible answers being progressive disease, stable disease, and partial/complete response. (**B**) Setting 2—In this setting, the binary segmentation masks are overlaid over the images. All metastases that the deep learning-based segmentation model found are indicated in orange. The overlay can be turned off by clicking the check box “Show lesions”. Information on overall hepatic tumor load is displayed on the right, above the treatment response evaluation. (**C**) Setting 3—On the left, the baseline and follow-up images can be found. The deep learning-based segmentation is overlaid and color-coded according to the categories in Table 1. If a metastasis grew more than 20%, it is labeled as progressive and is displayed in orange in the overlay. If it is not, the overlay is blue. The same color-coding is used in the table on the right panel. The metastases are numbered. Each metastasis has the same number at baseline and follow-up. The table shows the differential growth, with each row corresponding to an individual metastasis. The volume and diameter at both timepoints, as well as the absolute and relative difference between both timepoints, are shown. Above the table, the overall hepatic tumor load and the number of metastases per category are displayed. The table can be scrolled if it is too long to be displayed on the screen in full length.

Setting 1 only showed baseline and follow-up MRI (Figure 3A). Treatment response evaluation was based solely on the visual comparison of the two timepoints. In setting 2, automatic binary segmentation masks, which highlight the metastases, were overlaid on the MRI images (Figure 3B). This overlay could be turned off with a check box. Additionally, information about the hepatic tumor load was provided. Setting 3 included the most detailed information. Along with the MRI scans and segmentation masks, the results from the automatic 3D tracking were presented (Figure 3C). This included information about overall hepatic tumor load and detailed data on individual metastasis growth, such as volume and diameter changes. Furthermore, each metastasis was assigned to a diagnostic category (see Table 1), and the number per category was counted. New metastases and metastases with the largest growth were at the top of the table.

### 2.4. Statistical Analyses

The performance of the segmentation models was assessed using the Dice similarity coefficient [15], displaying the mean and standard deviation (SD). The normality of the data was examined using the Shapiro–Wilk test [16]. A significance level of *p* < 0.05 was applied for all tests. The Friedman test [17] was used for the group comparisons, with the pairwise Wilcoxon signed-rank test [18] with Bonferroni correction for multiple comparisons as a post hoc analysis. The predictive performance was evaluated using accuracy, precision, and recall.

## 3. Results

**Figure 4:**
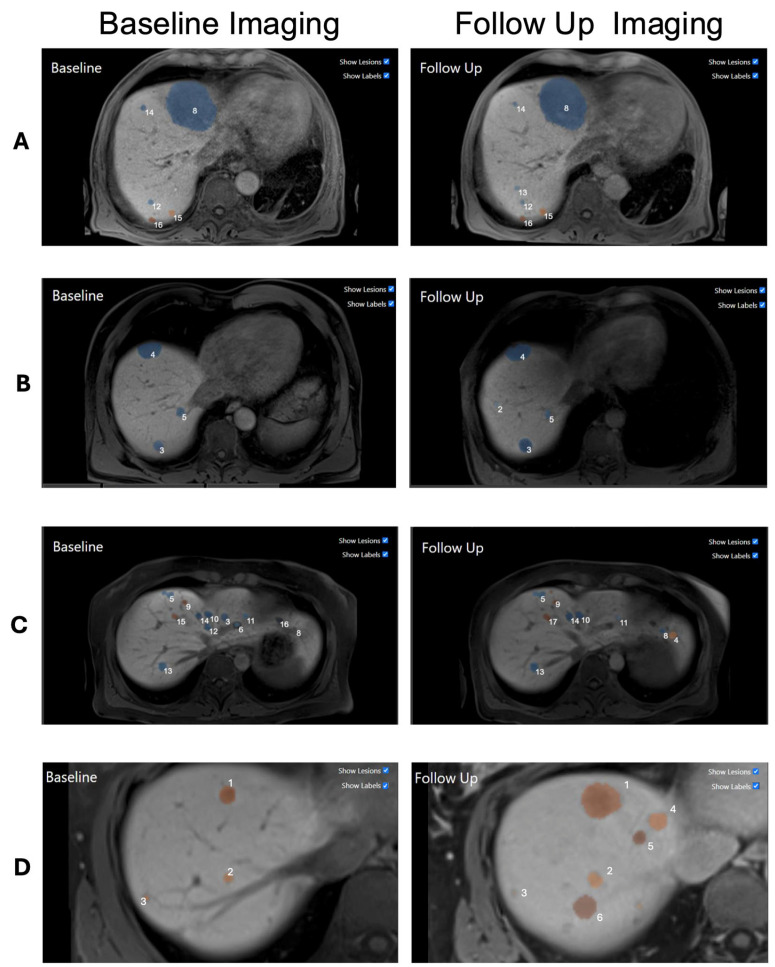
Example images for the 3D tracking results: In the left column, baseline MRIs are displayed, and in the right column are the corresponding follow-ups. The metastases are consistently labeled between the two timepoints. If a metastasis grew more than 20% or was new, it is color-coded in orange; if not, it is coded in blue. The 20% threshold is based on the RECIST guidelines, where it is applied to the sum of diameters of the target lesions. For individual metastases, no threshold is defined. For small lesions the 20% threshold does not seem to be ideal, as can be seen in the first example: (**A**) One large metastasis can be seen that remained stable between both timepoints. Additionally, five small metastases were found, two of which qualified for the label progressive with the 20% threshold. However, the true diagnosis is stable disease. (**B**) Example with four stable metastases. All four metastases remained stable, which matches with the diagnosis of stable disease. (**C**) Example with many metastases that have varying growth rates. All of them are small at both timepoints. Here, the MTB decision was stable disease. (**D**) Example of progressive disease. The three metastases already present in the baseline all grew more than 20%. Additionally, there were three new metastases in the follow-up.

Our algorithm detected 991 individual metastases in the 30 patients of the experimental cohort, 13% of which were new (n = 127), 30% progressive (n = 293), 18% stable (n = 180), and 18% regressive (n = 179). The remaining metastases were either smaller than 5 mm in diameter (15%; n = 151) or merged with another metastasis in the follow-up assessment (6%; n = 61). The segmentation model reached a mean Dice similarity coefficient of 0.83 (SD 0.11) in detecting NELMs compared to the ground-truth manual segmentations. Examples of the tracking algorithm can be seen in Figure 4.

**Figure 5:**
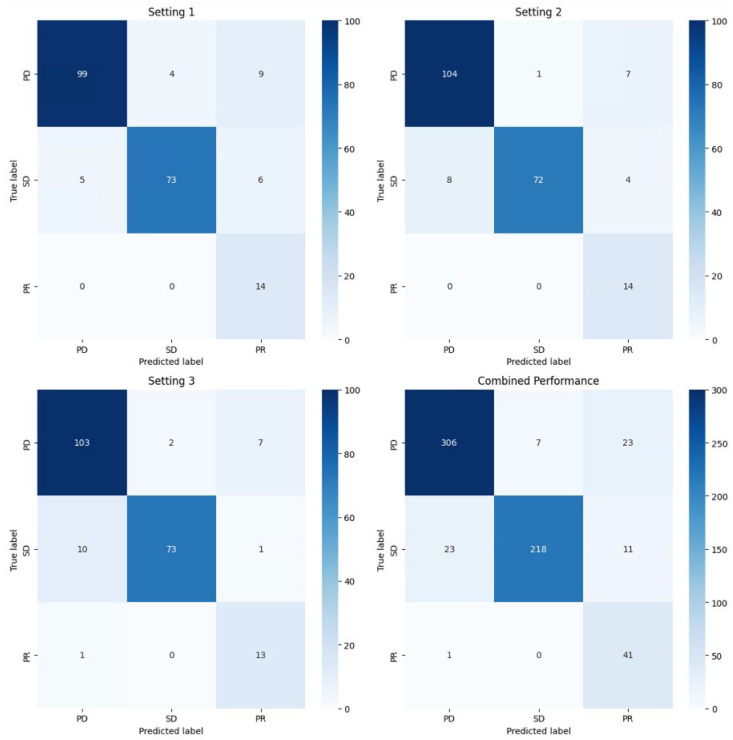
Confusion matrices for the three diagnostic settings and the combined performance over all settings. Assessments of 7 radiologists were compared to the ground truth for 30 cases, resulting in 210 evaluations per setting and 630 in total. Setting 1 included only baseline and follow-up MRI. Setting 2 added information on hepatic tumor load using deep learning-based segmentation of NELMs. Setting 3 included tumor load, metastasis categorization, and differential growth of each metastasis. The matrices display results for PD (progressive disease), SD (stable disease), and PR (partial response).

In the user study, the average accuracy of the response evaluation (progressive disease, stable disease, partial response) was lowest in setting 1, in which no additional information was provided, with 88.7% (precision 81.6%; recall 91.9%). In setting 2—where information about overall hepatic tumor load was added—the average accuracy was 90.6% (precision 83.6%; recall 92.9%). In setting 3—in which information of differential growth was included—the accuracy was 90.1% (precision 83.4%; recall 90.7%). However, the results were not significantly different between the three settings (*p* = 0.72). The results are summarized in Table 2. The performance per setting is further visualized in the confusion matrices in Figure 5.

**Figure 6:**
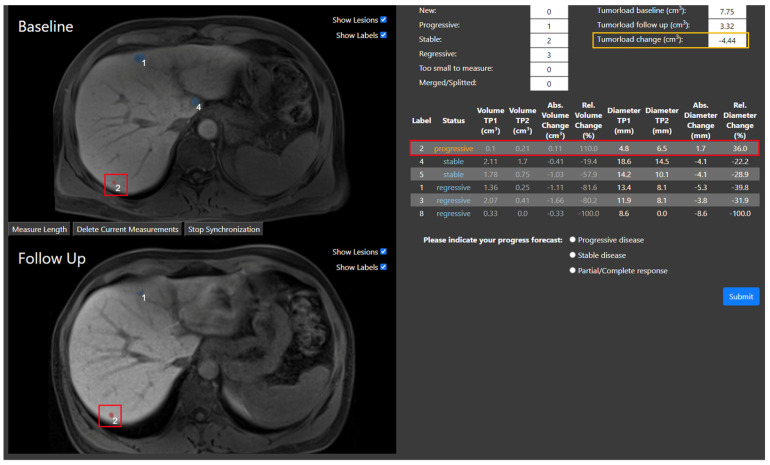
Example case in which the ground truth was progressive disease, but the overall hepatic tumor load decreased (orange rectangle) between the two timepoints, and only an individual metastasis grew, qualifying for the status of progressive disease. The metastasis has the label 2 and is marked with a red rectangle in the baseline and follow-up MRIs. The corresponding row in the differential growth table is also marked with a red rectangle. The automatic 3D tracking algorithm correctly identified this metastasis. However, the radiologists still chose either partial response or stable disease.

Five additional progressive cases were correctly identified in setting 2, and four in setting 3, compared to setting 1. One case was misclassified by all participants in all three settings. This makes up one-third of all errors in the study. Although the automatic 3D tracking algorithm correctly identified and displayed a progressive metastasis, six radiologists selected partial response and one selected stable disease as their prognosis (see Figure 6). The diameter of the progressive metastasis increased by 36%, from 4.8 mm to 6.5 mm. Meanwhile, the overall tumor load decreased by 4.44 cm^3^, as the other five metastases decreased in size.

Throughout the user study, the decision time per case was measured. The median decision time was 13.8 s for setting 1, 14.4 s for setting 2, and 23.8 s for setting 3 (Table 2). The Friedman test revealed a significant difference between the groups (*p* < 0.001, test statistic = 77.75). Post hoc tests showed that the diagnostic time was significantly longer in setting 3 compared to settings 1 and 2 (adjusted *p* < 0.001 in both comparisons), and in setting 2 compared to setting 1 (adjusted *p* = 0.01).

The radiologists indicated that they were certain in their decision in 94% of the cases, including the case described in Figure 6. There was no difference in certainty between the different settings.

The final survey revealed that the radiologists mostly valued the automatic calculation of hepatic tumor load and the overlay of the color-coded segmentation masks. In the comments, all seven radiologists noted that the segmentations were not always perfect, leading to occasional false calculations and mistrust. This cautious response to the apparent segmentation errors prompted the radiologists to verify the AI outputs before accepting them. We consider this practice to be essential for the responsible use of AI-generated information in clinical settings, although it led to an increase in diagnosis time. Despite this, the overall feedback was positive. All raters agreed that the tool was useful and that they would use a tool like this in their clinical practice. However, the radiologists expressed a desire for integration with other systems, and one participant indicated that they had difficulties working with the tool. The questions and answers from the final survey can be found in Appendix A.

## 4. Discussion

In this study, we developed a method to automatically track the differential growth of neuroendocrine liver metastases (NELMs). Our 3D tracking algorithm uses deep learning-based segmentations, enabling a time-efficient evaluation process that eliminates the need for manual segmentations. The key innovation of our work is the ability to isolate individual metastases from the segmentation masks, which allows us to extract data on the volume and diameter of individual metastases and compare these values between baseline and follow-up assessments. To test the usefulness of this information, we conducted a user study with an expert group of radiologists.

The user study demonstrated that diagnostic accuracy can be improved—albeit not significantly—by providing automatically generated information about hepatic tumor load and differential growth. Importantly, the number of correctly identified progressive cases increased. Detecting cancer progression early is important for timely treatment adjustments and may improve outcomes and quality of life for patients. In one case, the tool correctly identified a single progressive metastasis, but none of the participating radiologists classified the case as progressive disease. This raises concerns about the level of trust in automatically generated diagnostic information. The decision time increased with increasing amounts of information. A probable reason for this is that the radiologists had to process more information and compare it with their own assessments.

There is an ongoing debate about the use of unidimensional and bidimensional measurements, such as RECIST, as they may not fully account for irregular tumor shapes or heterogeneous growth patterns [19,20,21]. Volumetric approaches can provide a more accurate reflection of tumor burden and treatment effect. This shift towards volumetric assessment could mark a paradigm change in clinical practice, particularly as deep learning-based segmentations are advancing and reducing the burden of manual segmentation.

For example, in a previous study, NELMs were segmented automatically with high accuracy, and tumor load quantification closely matched human evaluations [12]. However, this approach did not account for the differential growth of individual tumors. Similarly, other studies that aimed to automatically monitor tumor treatment response did not account for individual growth rates. For instance, a study evaluated the potential of longitudinal analysis of glioblastoma response assessment from automatic tumor segmentation [22]. Another example is a neuro-oncology study that tracked tumor progression over time and, although it distinguished individual tumors, this information was used to identify new lesions rather than to calculate the individual tumor growth. Instead, the authors used cumulative bounding-box volumes to predict the time to progression [23]. A tool for the automatic quantification of tumor growth rate from follow-up CT has been developed for non-small-cell lung cancer. While this study analyzed individual tumor growth over several subsequent scans, the calculation was limited to a single, manually selected target lesion [24].

Few studies exist that include longitudinal tracking of individual lesions. One study developed a post-processing method that quantified how multiple sclerosis lesions change over time [25]. Similarly, another study focused on tracking individual brain metastases after stereotactic radiosurgery [26]. These solutions follow a similar methodology to our study but are tailored to brain imaging and different clinical endpoints. Thus, our study addresses a research gap by developing a method for liver imaging, specifically for NELMs.

The limitations of this study include the retrospective nature of the data collection, which may introduce selection bias. Additionally, the data were sourced from a single clinic, potentially restricting the generalizability of the findings. However, the inclusion of an additional dataset and the use of various scanners within the clinic strengthen this study by providing a broader context for validating the results. Specifically, the segmentation model might benefit from training with a larger, multi-center dataset. Another limitation is the small number of radiologists in the expert group, with some having limited experience in abdominal imaging, which may affect the generalizability of the findings. However, the results offer preliminary insights into the usefulness of the interface and can serve as a basis for further refinement and development.

## 5. Conclusions

Three-dimensional (3D) tracking can be an additional source of information for radiologists to observe tumor progression. Especially in cases with many lesions, this can prevent oversights of individually growing lesions. Moving forward, the focus should be on enhancing the segmentation model by using a large, multi-center dataset and integrating the tool into existing diagnostic tools to facilitate its practical application.

## Figures and Tables

**Table 1:** Classes of metastasis adapted from the response evaluation criteria in solid tumors (RECIST1.1) guidelines.

Name	Definition
Progressive	The diameter increased by at least 20%
Stable	The diameter change is between 20% increase and 30% decrease
Regressive	The diameter decreased by at least 30%
New	The lesion only appears in the follow-up MRI
Merged	The lesion grew together with another lesion
Too small to measure	The lesion has a diameter smaller than 5 mm at both timepoints

**Table 2:** Results per setting: The table shows the median decision time and the performance metrics for the treatment response evaluation, including accuracy, precision, and recall. PD = progressive disease, SD = stable disease, PR/CR = partial response/complete response, SD = standard deviation, IQR = interquartile range.

Metric	Setting 1	Setting 2	Setting 3	*p*-Value
Median decision time in s (IQR)	13.8 (9.2–21.8)	14.4 (10.3–24.0)	23.8 (14.2–42.8)	<0.001
Accuracy in % (SD, range)	88.7 (SD 11.0, range 67–97)	90.6 (SD 8.7, range 73–97)	90.1 (SD 6.1, range 80–97)	0.72
Precision in % (SD, range)	81.6 (SD 9.5, range 63–89)	83.6 (SD 6.4, range 72–89)	83.4 (SD 5.8, range 73–89)	0.72
Recall in % (SD, range)	91.9 (SD 8.7, range 74–98)	92.9 (SD 7.2, range 78–98)	90.7 (SD 9.4, range 71–98)	0.30

**Figure A1:**
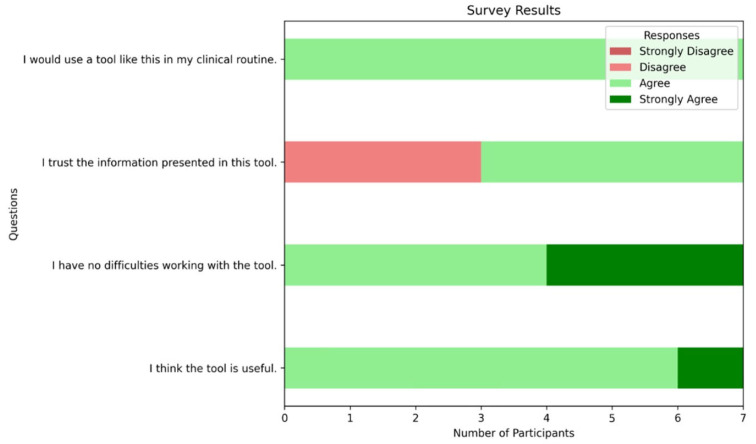
Responses to the survey: After completing all cases, the participants were asked four general questions regarding the tool. The possible answers were on a four-item Likert Scale: strongly disagree, disagree, agree, and strongly agree.

**Figure A2:**
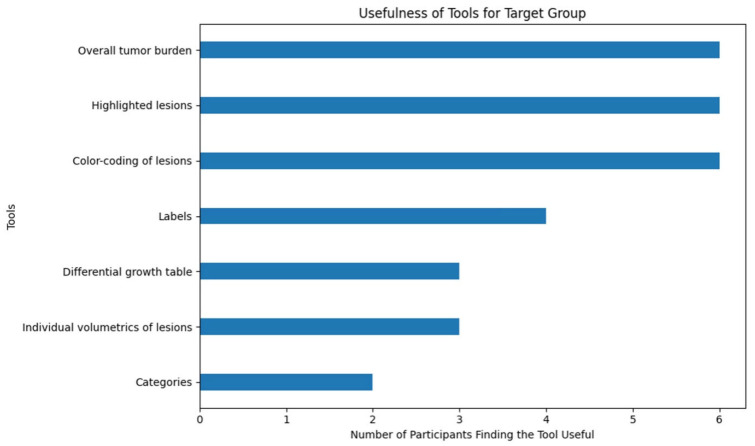
Responses to the usefulness of the individual parts of the interface. Participants were encouraged to select all that apply.

## Data Availability

The datasets generated and analyzed during the current study are not publicly available, due to privacy concerns, but are available from the corresponding author upon reasonable request.

## Abbreviations

The following abbreviations are used in this manuscript:

FS	Fat saturation
Gd-EOB	Gadoxetic acid
GRE	Gradient echo
HBP	Hepatobiliary contrast phase
MTB	Multidisciplinary Tumor Board
MRI	Magnetic resonance imaging
NELM	Neuroendocrine liver metastasis
RECIST	Response evaluation criteria in solid tumors
SD	Standard deviation
VIBE	Volumetric interpolated breath-hold examination

## Appendix A. Survey Results

### Appendix A.1. Survey Questions

After completion of the user study, the radiologists were asked to rate how much they agree with the following statements on a four-item Likert Scale (strongly disagree, disagree, agree, and strongly agree). The results can be seen in Figure A1.

1. I think the tool is useful.
2. I have no difficulties working with the tool.
3. I trust the information presented in this tool.
4. I would use a tool like this in my clinical routine.

Additionally, they were asked to indicate which information they found helpful. They were encouraged to select all that apply. The results can be seen in Figure A2.

- Overall tumor burden
- Highlighted lesions
- Color-coding of lesions
- Showing the unique labels
- Differential growth table
- Individual volumetrics of lesions
- Categories

Lastly, they were encouraged to provide feedback in written form as a comment.
